# Visualization of small brain nuclei with a high-spatial resolution, clinically available whole-body PET scanner

**DOI:** 10.1007/s12149-023-01886-1

**Published:** 2023-11-21

**Authors:** Yuki Shinohara, Masanobu Ibaraki, Keisuke Matsubara, Kaoru Sato, Hiroyuki Yamamoto, Toshibumi Kinoshita

**Affiliations:** 1 Department of Radiology and Nuclear Medicine, Research Institute for Brain and Blood Vessels-Akita, 6-10 Senshu-kubota-machi, Akita, 010-0874 Japan; 2 Department of Management Science and Engineering, Faculty of System Science and Technology, Akita Prefectural University, Yurihonjo, Japan

**Keywords:** ^18^F-fluorodeoxyglucose, Whole-body silicon photomultiplier positron emission tomography, Point-spread function, Iteration, Three-dimensional fluid-attenuated inversion recovery image

## Abstract

**Objective:**

To verify the visibility of physiological ^18^F-fluorodeoxyglucose (^18^F-FDG) uptake in nuclei in and around the brainstem by a whole-body (WB) silicon photomultiplier positron emission tomography (SiPM-PET) scanner with point-spread function (PSF) reconstruction using various iteration numbers.

**Methods:**

Ten healthy subjects (5 men, 5 women; mean age, 56.0 ± 5.0 years) who underwent ^18^F-FDG PET/CT using a WB SiPM-PET scanner and magnetic resonance imaging (MRI) of the brain including a spin-echo three-dimensional sampling perfection with application-optimized contrasts using different flip angle evolutions fluid-attenuated inversion recovery (3D-FLAIR) and a 3D-T1 magnetization-prepared rapid gradient-echo (T1-MPRAGE) images were enrolled. Each acquired PET image was reconstructed using ordered-subset expectation maximization (OSEM) with iteration numbers of 4, 16, 64, and 256 (subset 5 fixed) + time-of-flight (TOF) + PSF. The reconstructed PET images and 3D-FLAIR images for each subject were registered to individual T1-MPRAGE volumes using normalized mutual information criteria. For each MR-coregistered individual PET image, the pattern of FDG uptake in the inferior olivary nuclei (ION), dentate nuclei (DN), midbrain raphe nuclei (MRN), inferior colliculi (IC), mammillary bodies (MB), red nuclei (RN), subthalamic nuclei (STN), lateral geniculate nuclei (LGN), medial geniculate nuclei (MGN), and superior colliculi (SC) was visually classified into the following three categories: good, clearly distinguishable FDG accumulation; fair, obscure contour of FDG accumulation; poor, FDG accumulation indistinguishable from surrounding uptake.

**Results:**

Among individual ^18^F-FDG PET images with OSEM iterations of 4, 16, 64, and 256 + TOF + PSF, the iteration numbers that showed the best visibility in each structure were as follows: ION, MRN, LGN, MGN, and SC, iteration 64; DN, iteration 16; IC, iterations 16, 64, and 256; MB, iterations 64 and 256; and RN and STN, iterations 16 and 64, respectively. Of the four iterations, the ^18^F-FDG PET image of iteration 64 visualized FDG accumulation in small structures in and around the brainstem most clearly (good, 98 structures; fair, 2 structures).

**Conclusions:**

A clinically available WB SiPM-PET scanner is useful for visualizing physiological FDG uptake in small brain nuclei, using a sufficiently high number of iterations for OSEM with TOF and PSF reconstructions.

**Supplementary Information:**

The online version contains supplementary material available at 10.1007/s12149-023-01886-1.

## Introduction

The brainstem nuclei and their neural networks play pivotal roles in life support, including regulating consciousness, respiration, and circulation. In addition, the brainstem has multiple nuclei and complex neural networks that connect with other structures in the brain including the diencephalon and cerebellum. It is well known that neurodegenerative diseases, psychiatric disorders, and secondary degeneration resulting from cerebrovascular disease are closely related to abnormalities of the nuclei and neural networks in the brainstem [1].

Several articles have reported recent advances in radiographic technology that have improved anatomical microstructural visualization of the brain, such as advanced magnetic resonance imaging (MRI) sequences that can depict the subthalamic nucleus, nuclei of the brainstem, and corticospinal tract [2–4]. Remarkable progress has also been made in positron emission tomography (PET). A PET scanner developed with a silicon photomultiplier (SiPM) and time-of-flight (TOF) capability [5, 6] in combination with full 3D image reconstruction with resolution modeling (point-spread function [PSF] reconstruction) enables generation of high-resolution images [7–11]. Recent articles have indicated that ^18^F-fluorodeoxyglucose (^18^F-FDG) uptake in the small structures of the brain can be visualized using state-of-the-art, brain-dedicated SiPM-PET scanners [12–15] that outperform previous-generation brain-dedicated PET scanners, in terms of both spatial resolution and detection sensitivity [16, 17]. In our previous study, we compared the partial volume correction (PVC) performance of PSF reconstruction with MR-based PVC performance for measurements of FDG uptake in the cerebral cortex [11]. To the best of our knowledge, however, there is no previous report on a normal FDG uptake pattern of the brainstem nuclei using a clinically available whole-body (WB) SiPM-PET scanner.

We hypothesized that WB SiPM-PET with an image reconstruction setting optimized for brain would enable accurate identification of FDG uptake in the brainstem nuclei and its surrounding small structures. The aim of this study is to examine the detectability of physiological FDG uptake in nuclei in and around the brainstem on high resolution WB SiPM-PET (Biograph Vision, Siemens Healthineers, Knoxville, TN, USA) with PSF reconstruction using various numbers of iterations, in reference to the anatomical location on spin-echo three-dimensional sampling perfection with application-optimized contrasts using different flip angle evolutions fluid-attenuated inversion recovery (3D-FLAIR) imaging.

## Materials and methods

### Subjects

Ten healthy volunteers (5 males and 5 females; mean age ± standard deviation, 56.0 ± 5 years) who underwent ^18^F-FDG PET using WB SiPM-PET/CT (Biograph Vision, Siemens Healthineers) and MRI (MAGNETOM Skyra 3T, Siemens Healthineers) of the brain, the same cohort as in our previous study [11], were included. While the previous study investigated the FDG uptake in cerebral cortex with volume-of-interest analysis as the comparative study to MR-based PVC [11], in this study, we focused on the visual assessment of the uptake in small nuclei in and around the brainstem; the PET and MRI data acquired in the previous study were used. The imaging parameters used for WB SiPM-PET/CT (Biograph Vision, Siemens Healthineers) were as follows: transverse and axial spatial resolution, 3.6 and 3.5 mm, respectively; axial field of view, 26 cm; TOF resolution, 210 ps [5]. The subjects were determined as healthy based on their medical history and the MRI findings. The study protocol was approved by the ethics committee of our institution and written informed consent was obtained from each participant prior to the imaging examinations. The study was performed in accordance with the ethical standards laid down in the 1964 Declaration of Helsinki and all subsequent revisions.

### ^18^F-FDG PET

Each participant was instructed to avoid strenuous exercise for 24 h and fast for at least 4 h before 20-s intravenous injection of ^18^F-FDG (229 ± 16 MBq), which is the standard protocol for clinical ^18^F-FDG PET examination in our institution. To minimize head movement during the scan, the participants were asked to keep their eyes open and in resting condition, and the head was immobilized using pads and a Velcro band around the head and head holder. A 30-min PET list-mode acquisition was started 30 min after the injection, resulting in sufficiently high statistics of 950 ± 185 million coincidences (true plus scatter). A standard low-dose CT scan (120 kV, 100 mAs) was acquired prior to the PET acquisition for attenuation correction.

The list-mode data were reconstructed into single static images (30-min duration). The reconstruction algorithm was a 3D ordinary Poisson OSEM with TOF information and PSF modeling, termed PSF reconstruction, resulting in PET images with a 440 × 440 × 159 matrix (0.8 × 0.8 × 1.6 mm; post-reconstruction zoom factor, 2) [5, 18]. Iterations varied over a wide range, from 4 to 256 (5 subsets fixed). The setting recommended by the vendor is three to four iterations for WB ^18^F-FDG acquisitions. All data were corrected for random coincidences, detector normalization, radioactive decay, dead time count losses, scatter coincidences (single scatter simulation with the scaling option), and attenuation during the reconstruction. We used the reconstruction research software provided by the vendor (Siemens Healthineers). Computational time ranged from approximately 1 min for 4 iterations to 25 min for 256 iterations (Intel Core i7-9800X CPU 3.80 GHz and 128 GB memory). No post-reconstruction image filtering was applied.

### MRI

All participants underwent MRI of the brain on the day before PET, using a 3-T MRI scanner. The protocol included a 3D-FLAIR sequence and a 3D-T1 magnetization-prepared rapid gradient-echo (T1-MPRAGE) sequence. The scanning parameters for the two sequences were as follows. 3D-FLAIR: voxel size, 0.6 × 0.6 × 1.0 mm; sagittal slices; TR, 6000 ms; TE, 237 ms; TI, 2000 ms; FOV, 230 mm; turbo factor, 160; echo train duration, 492 ms; variable flip angle (T2 var mode); band width, 789 Hz/pixel; PAT mode, GRAPPA; acquisition time, 9 min 32 s; and T1-MPRAGE: voxel size, 0.8 mm; matrix size, 320 × 320 × 208; sagittal slices; TR, 2300 ms; TE, 3 ms; IR, 900 ms; flip angle, 9 degrees; distortion correction, 3D; acquisition time, 7 min 21 s.

### Image registration between PET and MR

The reconstructed PET images for each participant were registered to individual T1-MPRAGE volumes (non-brain tissues were stripped) using normalized mutual information criteria. All subsequent image processing was performed using the MR-registered PET images.

### Generation of average PET and MR maps using anatomical standardization

Before assessing FDG uptake in and around the brainstem on the individual PET images (i.e., single PET image), anatomical standardization was applied to generate average PET and MR maps across the bilateral hemispheres from all subjects (i.e., 20 hemispheres from 10 subjects). The aim of this process was to generate less-noisy average maps that were used to confirm anatomical correspondence between the observed FDG uptake and the low intensity seen on 3D-FLAIR. Anatomical standardization was performed using individual T1-MPRAGE images and Diffeomorphic Anatomical Registration using Exponentiated Lie algebra (DARTEL toolbox in Statistical Parametric Mapping 12 [SPM12] software package [https://www.fil.ion.ucl.ac.uk/spm/software/spm12/]) with the left–right symmetric version, according to Kurth et al. [19], and subsequently applied to the individual PET and 3D-FLAIR images. Anatomically standardized individual PET and MR images were further intensity-normalized using a gray-matter mask, duplicated with left–right flipping, and averaged, resulting in left–right symmetric average maps (*n* = 20 hemispheres).

**Fig. 1:**
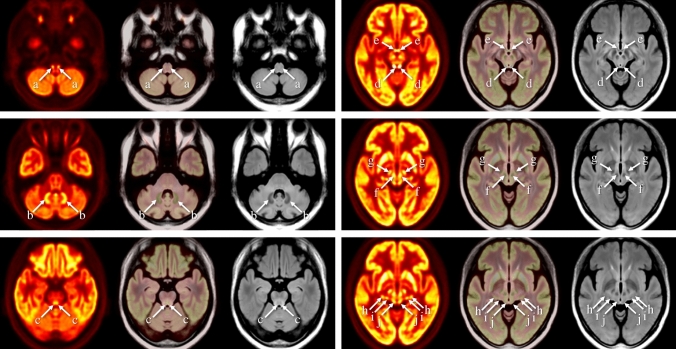
Average ^18^F-FDG PET (OSEM iteration 64 + TOF + PSF, left in each row), ^18^F-FDG PET/3D-FLAIR fusion (middle in each row), and 3D-FLAIR images (right in each row) (across hemispheres, *n* = 20). The average ^18^F-FDG PET image with OSEM iteration 64 + TOF + PSF reconstruction clearly shows FDG uptake in the olivary nuclei (a), dentate nuclei (b), midbrain raphe nuclei (c), inferior colliculi (d), mammillary bodies (e), red nuclei (f), subthalamic nuclei (g), lateral geniculate nuclei (h), medial geniculate nuclei (i), and superior colliculi (j)

Figure 1 and supplementary Fig. 1 shows the set of average maps for PET with 64 iterations, 3D-FLAIR, and fusion of these two maps. Based on the anatomical location [20–22] and signal intensity on the average MR maps, normal FDG uptake in and around the brainstem could be identified in the inferior olivary nuclei (ION), dentate nuclei (DN), midbrain raphe nuclei (MRN), inferior colliculi (IC), mammillary bodies (MB), red nuclei (RN), subthalamic nuclei (STN), lateral geniculate nuclei (LGN), medial geniculate nuclei (MGN), and superior colliculi (SC) on the average PET maps (Fig. 1 and supplementary Fig. 1). These ten nuclei were set as the target regions in subsequent visual assessment of FDG uptake in the individual PET images.

### Visual assessment on individual PET Images

For the MR-coregistered, individual ^18^F-FDG PET images applying OSEM iterations 4, 16, 64, and 256 (subset 5 fixed) with TOF and PSF reconstructions, the visibility of FDG uptake in the ten structures in each subject was classified as one of the following three categories by a neuroradiologist (YS, with 19 years of experience in neuroradiology) who also specializes in nuclear medicine: good, clearly distinguishable ^18^F-FDG accumulation; fair, obscure contour of ^18^F-FDG accumulation; poor, ^18^F-FDG accumulation indistinguishable from surrounding uptake.

## Results

**Fig. 2:**
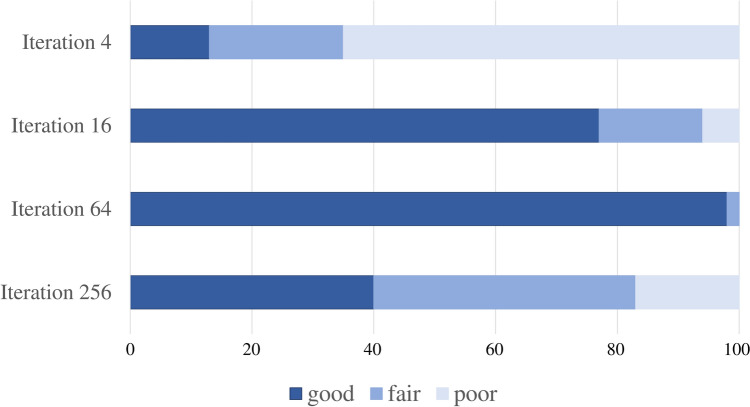
Visual assessment of individual ^18^F-FDG PET images with OSEM iterations 4, 16, 64, and 256 + TOF + PSF reconstruction. Among 100 structures (10 subjects × 10 structures in each) on ^18^F-FDG PET images using OSEM + TOF + PSF, iteration 64 was “good” in 98 and “fair” in 2; iteration 16 was “good” in 77, “fair” in 17, and “poor” in 6; iteration 256 was “good” in 40, “fair” in 43, and “poor” in 17; and iteration 4 was “good” in 13, “fair” in 22, and “poor” in 65

Among all of the 100 structures (10 subjects × 10 structures in each), the ^18^F-FDG PET findings for each iteration were as follows: iteration 64, “good” in 98 and “fair” in 2; iteration 16, “good” in 77, “fair” in 17, and “poor” in 6; iteration 256, “good” in 40, “fair” in 43, and “poor” in 17; and iteration 4, “good” in 13, “fair” in 22, and “poor” in 65 (Fig. 2).

Among each structure, the ^18^F-FDG PET findings for each iteration were as follows.

1. ION: iteration 64, “good” in 10; iterations 256 and 16, “good” in 8 and “fair” in 2; iteration 4, “fair” in 1 and “poor” in 9.
2. DN: iteration 16, “good” in 10; iteration 64, “good” in 9 and “fair” in 1; iteration 4, “good” in 5 and “fair” in 5; iteration 256, “good” in 3, “fair” in 6 and “poor” in 1.
3. MRN: iteration 64, “good” in 9 and “fair” in 1; iteration 16, “good” in 3 and “fair” in 7; iteration 256, “good” in 3, “fair” in 4 and “poor” in 3; iteration 4, “fair” in 2 and “poor” in 8.
4. IC: iterations 256, 64, and 16, “good” in 10; iteration 4, “good” in 7 and “fair” in 3.
5. MB: iterations 256 and 64, “good” in 10; iteration 16, “good” in 7 and “fair” in 3; iteration 4, “fair” in 1 and “poor” in 9.
6. RN: iterations 64 and 16, “good” in 10; iteration 256, “good” in 1, “fair” in 8 and “poor” in 1; iteration 4, “good” in 1, “fair” in 7 and “poor” in 2.
7. STN: iterations 64 and 16, “good” in 10; iteration 256, “good” in 1, “fair” in 8 and “poor” in 1; iteration 4, “fair” in 2 and “poor” in 8.
8. LGN; iteration 64, “good” in 10; iteration 16, “good” in 5, “fair” in 2 and “poor” in 3; iteration 256, “good” in 1, “fair” in 6 and “poor” in 3; iteration 4, “poor” in 10.
9. MGN: iteration 64, “good” in 10; iteration 16, “good” in 5, “fair” in 2 and “poor” in 3; iteration 256, “good” in 1, “fair” in 6 and “poor” in 3; iteration 4, “poor” in 10.
10. SC: iteration 64, “good” in 10; iteration 16, “good” in 9 and “fair” in 1; iteration 256, “good” in 2, “fair” in 3 and “poor” in 5; iteration 4, “fair” in 1 and “poor” in 9 (supplementary Table 1 and supplementary Fig. 2).

Thus, the number of iterations that demonstrated the best visibility in each structure were as follows: ION, MRN, LGN, MGN, and SC, iteration 64; DN, iteration 16; IC, iterations 16, 64, and 256; MB, iterations 64 and 256; and RN and STN, iterations 16 and 64, respectively.

**Fig. 3:**
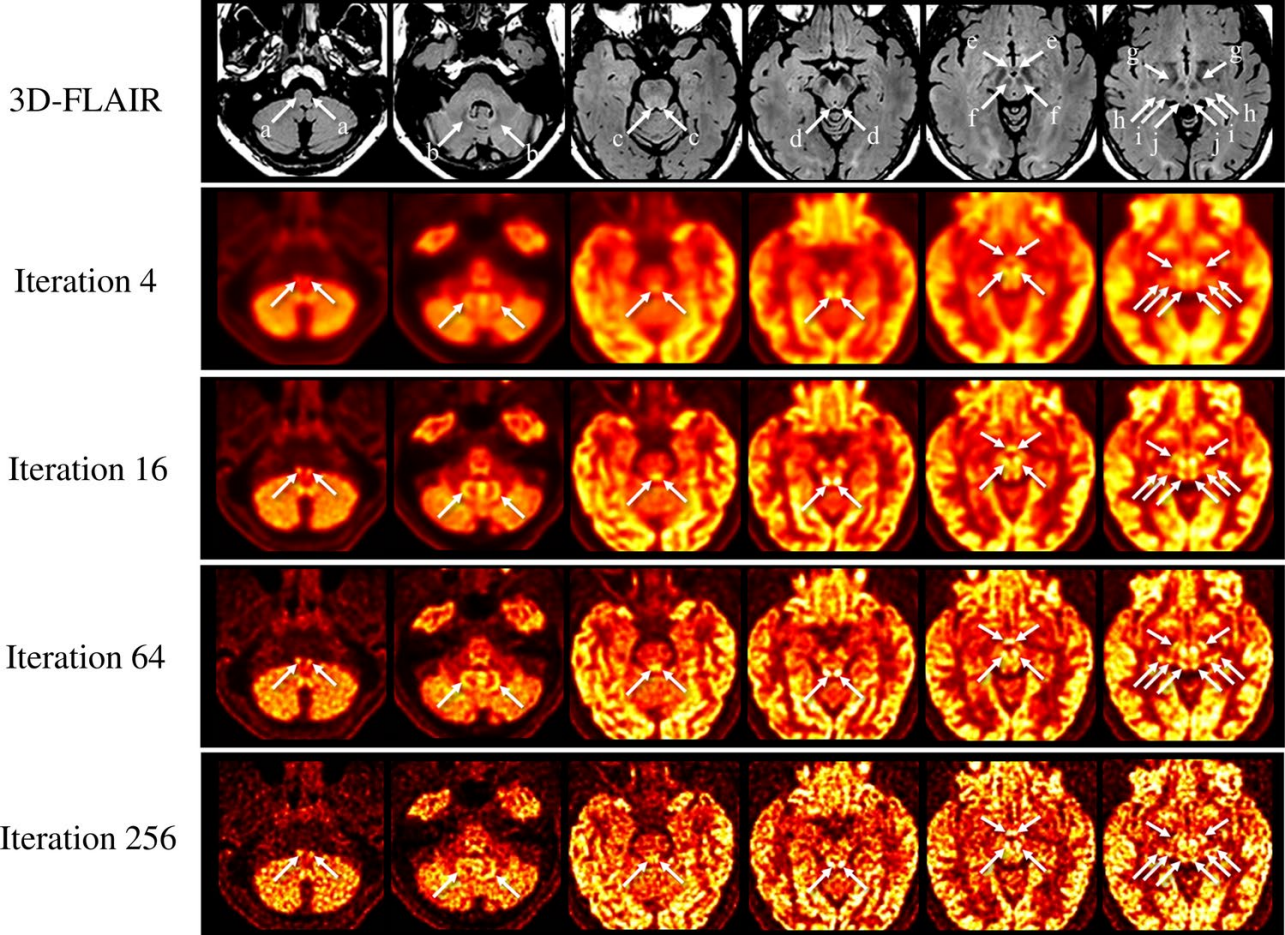
Individual 3D-FLAIR image (first row) and ^18^F-FDG PET images with OSEM + TOF + PSF reconstruction (second to fifth rows) in a representative participant. On the individual ^18^F-FDG PET image with OSEM iteration 64 + TOF + PSF (fourth row), FDG uptake can be clearly distinguished (good) in all structures, including the inferior olivary nuclei (ION, a), dentate nuclei (DN, b), midbrain raphe nuclei (MRN, c), inferior colliculi (IC, d), mammillary bodies (MB, e), red nuclei (RN, f), subthalamic nuclei (STN, g), lateral geniculate nuclei (LGN, h), medial geniculate nuclei (MGN, i), and superior colliculi (SC, j). On iteration 16 (third row), ^18^F-FDG accumulation can be clearly seen (good) in the DN (b), IC (d), RN (f), STN (g), and SC (j); and is obscure (fair) in the ION (a), MRN (c), MB (e), LGN (h), and MGN (i). On iteration 256 (fifth row), FDG uptake is good in the ION (a), DN (b), IC (d), MB (e), and SC (j); and fair in the MRN (c), RN (f), STN (g), LGN (h), and MGN (i). On iteration 4 (second row), ^18^F-FDG accumulation is fair in the DN (b), IC (d), and RN (f); and cannot be distinguished with surrounding uptake (poor) in other structures, including the ION (a), MRN (c), MB (e), STN (g), LGN (h), MGN (i), and SC (j)

Representative sections of ^18^F-FDG PET, fused PET/3D-FLAIR, and 3D-FLAIR images are shown in Fig. 3 and supplementary Fig. 3.

## Discussion

In this study, we verified normal FDG uptake of nuclei located in and around the brainstem using a clinically available, high-resolution WB scanner, a Biograph Vision PET/CT system (Siemens Healthineers). We found that a sufficiently high iteration number for OSEM, particularly iteration 64, with TOF and PSF reconstructions led to clear visualization of physiological FDG uptake in nuclei in and around the brainstem. It should be emphasized that when minimum iteration numbers were applied (iteration = 4), corresponding to the setting using for clinical WB ^18^F-FDG, visibility of FDG uptake was poor for most nuclei.

The brainstem nuclei and their neural networks perform a crucial role in regulating basic life-sustaining activities. Numerous previous articles have already reported that brainstem nuclei can be visualized using appropriate MRI sequences such as fast gray matter acquisition T1 inversion recovery, 3D-FLAIR, short tau inversion recovery, and so forth [2, 3, 23–27]. In this study, we used T1-MPRAGE and 3D-FLAIR as reference images for the averaged and individual ^18^F-FDG PET images to facilitate recognition of which tracer accumulation corresponded to which brainstem nucleus.

It has been considered that conventional WB PET scanners are inadequate to evaluate small brain structures, including brainstem nuclei, because of their relatively poor spatial resolution. Indeed, several reports have described PET-tracer uptake in the brainstem nuclei using head-dedicated PET scanners [12, 13, 16]. For example, Takahashi et al. described that FDG uptake in small brainstem nuclei such as the IC, RN, and MRN were more clearly detected by a brain-dedicated PET system with a hemispherical shape than by WB PET [12]. Thus, to the best of our knowledge, the present study is the first to clarify the utility of WB SiPM-PET with application of dedicated OSEM with TOF and PSF reconstruction to detect physiological FDG uptake in and around the brainstem (including ION, DN, MRN, IC, MB, RN, STN, LGN, MGN, and SC) with reference to the anatomical locations on 3D-MRI.

The result of this study reflects not only high resolution of WB SiPM-PET scanner itself but the intrinsic characteristics of PSF reconstruction including its relationship between number of iterations and acquired image quality [7–11]. That is, brain ^18^F-FDG PET with PSF reconstruction on WB SiPM-PET can provide high image contrast to visualize small structures in and around brainstem when applying sufficiently high iteration number. At the same time, however, our result also indicates that increasing iteration number too much has a risk to generate image noise, resulting in worsening the image quality [11].

At present, some studies have reported glucose metabolism in individual brainstem nuclei in relation to the brain functions. For instance, Hirata et al. clarified the relationship between FDG uptake in RN and metabolic activity of the cerebrum and cerebellum using a direct conversion semiconductor PET scanner [17]. The RN is one of the nuclei in the Guillain-Mollaret triangle, which contains an ipsilateral RN and ION in addition to a contralateral DN. The recent study by Speck et al. using a fully digital high-resolution PET system indicated that FDG uptakes in the IC and primary auditory cortex were correlated with asymmetric hearing loss [9]. It is known that the auditory pathway contains the MGN as well as IC in and around the brainstem. Furthermore, although sufficient spatial resolution could not always be obtained on the PET images, other previous studies have also investigated glucose metabolism in and around the brainstem in various types of neurodegenerative disease. Jaillard et al. presented a patient with progressive supranuclear palsy in which decreased FDG accumulation in the SC could be evaluated [28]. One case series, which included nine patients with palatal myoclonus and ION pseudohypertrophy, reported that decreased FDG uptake in the ipsilateral pontine tegmentum and increased FDG uptake in the contralateral thalamus were shown in 6 cases and FDG uptake in the ION was normal in all cases [29]; whereas Dubinsky et al. reported a case of palatal myoclonus with increased FDG uptake in ION [30]. Thus, visualization of small structures such as the RN, IC, ION, DN, MGN, and SC, which was possible in the present FDG-PET examinations using the WB SiPM-PET system with optimized reconstruction, may enable the acquisition of additional knowledge associated with these neural networks.

**Fig. 4:**
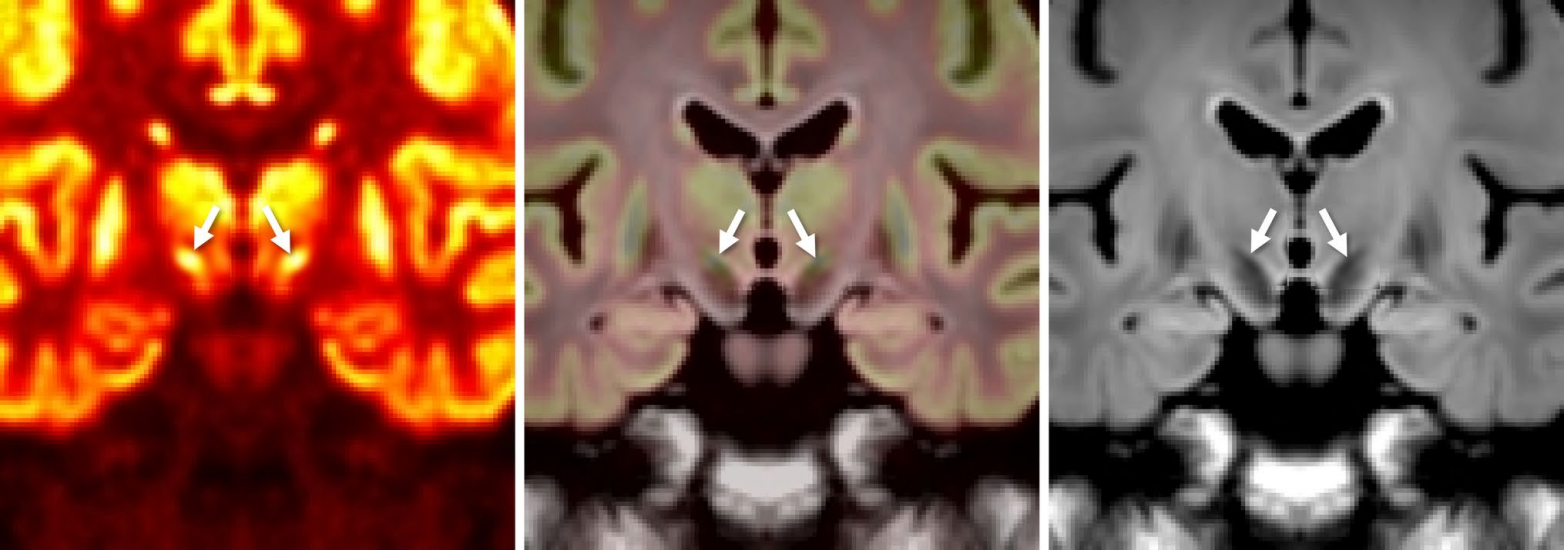
^18^F-FDG accumulation in subthalamic nuclei. Coronal average ^18^F-FDG PET image with reconstruction of OSEM iteration 64 + TOF + PSF (left, arrows) demonstrates avid FDG uptake corresponding to subthalamic nuclei (^18^F-FDG PET/3D-FLAIR fusion image, middle, arrows), which are well-recognized on coronal 3D-FLAIR images (right, arrows)

One of the novel findings in this study was physiological FDG uptake corresponding to the STN (Fig. 4). Although FDG accumulation in the substantia nigra is already known [16], this is the first study to demonstrate more avid physiological FDG uptake in the STN than that in the substantia nigra. The improved visualization is due not only to the high spatial resolution achieved by the WB SiPM-PET system and by applying a sufficiently high number of iterations for OSEM with TOF and PSF reconstructions, but also to the evaluation of FDG-PET images with reference to the 3D-FLAIR images, which are considered useful for identifying the STN [24, 25]. In the clinical setting, it is important for neurosurgeons to accurately identify the anatomical location of the STN on MRI because it is the most common target structure for deep brain stimulation, which is one of the treatment options for advanced Parkinson’s disease. Although the relationship between the degree of FDG uptake in the STN and the severity of Parkinson’s disease or the efficacy of STN stimulation remains unclear, it would be worth investigating in a future study using the high spatial resolution SiPM-PET scanner with an appropriate reconstruction method as described above.

This study has several limitations. First, the participants were a small number of healthy subjects. Second, we did not conduct a region-of-interest (ROI) analysis for quantitative measurement such as standardized uptake value (SUV). It was difficult to perform accurate ROI analysis in the acquired PET and MR images because, as far as we know, there is no suitable ROI template for the brainstem nuclei and its surrounding small structures based on the established anatomical atlas. Although a manual ROI placement with reference to the anatomical location on 3D-FLAIR image might be possible, it is quite subjective so that inaccurate ROI setting can cause measurement error of SUV. Quantitative or semiquantitative (e.g., SUV ratio) analysis with sophisticated ROI-based analysis using enough number of subjects, is therefore warranted. Third, the number of subsets for OSEM reconstruction was fixed at 5 in this study, which was the value recommended by the vender side. Since the parameter that determines the image contrast and noise characteristics is the update number (multiplication of subset and iteration numbers), it is also important to note the difference in the number of subsets when comparing the present study with other studies using different PET scanners. Finally, we did not perform motion correction during the PET data acquisition. Although motion-related artifact might have been negligible in this healthy-control study, evaluation of pathological conditions is required in a future study.

In conclusion, a clinically available WB SiPM-PET scanner was useful for visualizing physiological FDG accumulation in the brainstem nuclei of healthy subjects, using a sufficiently high number of iterations for OSEM with TOF and PSF reconstructions. WB SiPM-PET is expected to be valuable for functional evaluation of the brainstem and cerebellum in patients with neurodegenerative diseases, neuropsychiatric diseases, and cerebrovascular disorders including remote effects.

### Supplementary Information

Below is the link to the electronic supplementary material.

Supplementary file1 (PDF 725 KB)
