# Supplementary material for: Visualization of small brain nuclei with a high-spatial resolution, clinically available whole-body PET scanner
Source: Ann Nucl Med. 2023 Nov 21;38(2):154–61. doi: 10.1007/s12149-023-01886-1 (PMC10822807; doi:10.1007/s12149-023-01886-1)
Supplement: Supplementary file 1 — Supplementary file1 (PDF 725 KB) [file 12149_2023_1886_MOESM1_ESM.pdf]

Visualization of small brain nuclei with a high-spatial resolution, clinically available whole-body PET scanner

Annals of Nuclear Medicine

Yuki Shinohara, Masanobu Ibaraki, Keisuke Matsubara, Kaoru Sato, Hiroyuki Yamamoto, Toshibumi Kinoshita

Corresponding author: Yuki Shinohara, MD, PhD, Department of Radiology and Nuclear Medicine, Research Institute for Brain and Blood Vessels-Akita, 6-10 Senshu-kubota-machi, Akita 010-0874, Japan

Tel: +81-18-833-0115; Fax: +81-18-833-2104

Email: shino-y@akita-noken.jp

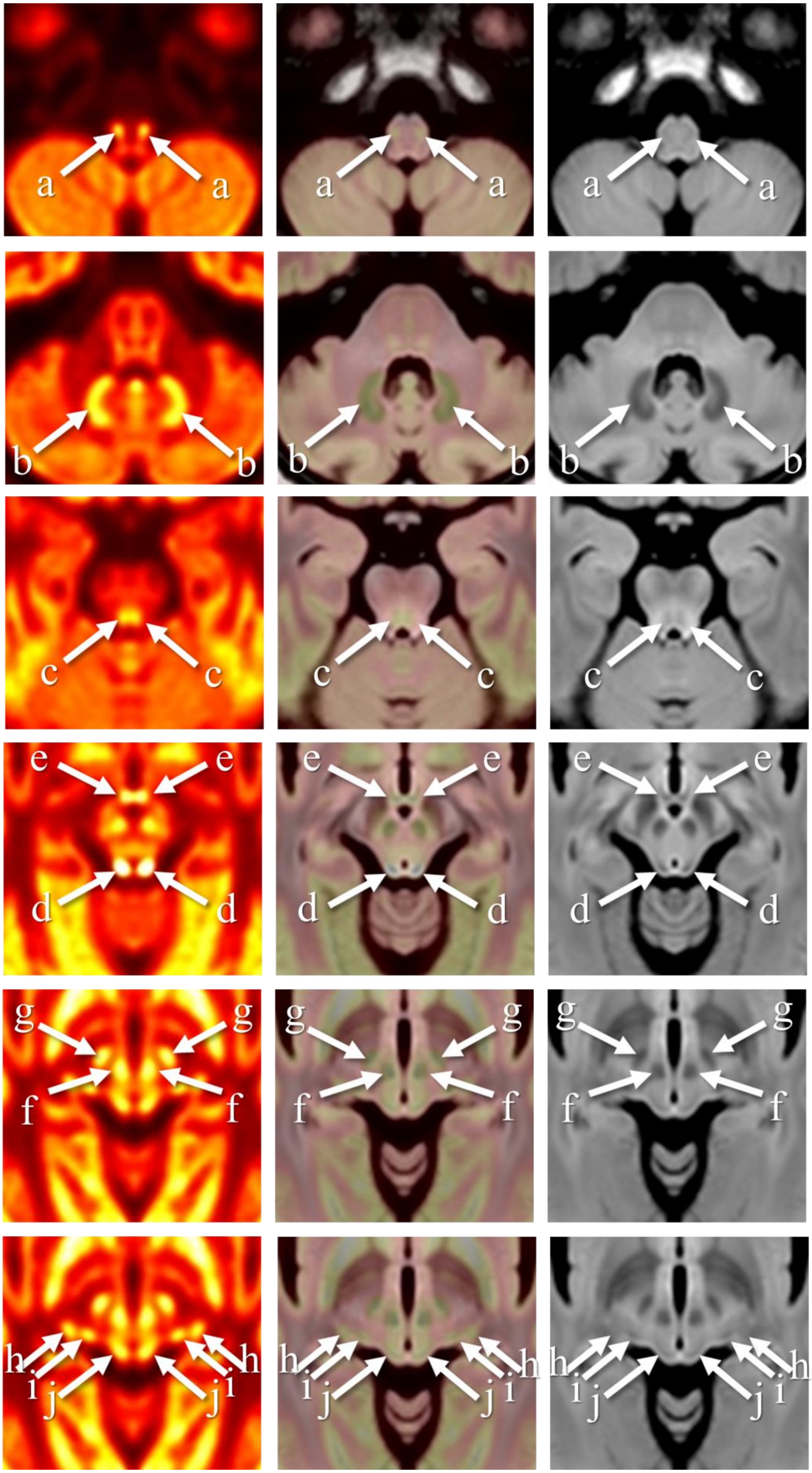

Supplementary Fig. 1. Zoomed average ^{18}F-FDG PET (OSEM iteration 64 + TOF + PSF, left row), ^{18}F-FDG PET/3D-FLAIR fusion (middle row), and 3D-FLAIR images (right row) (across hemispheres, n = 20).

The zoomed average ^{18}F-FDG PET image clearly shows FDG uptake in the inferior olivary nuclei (a), dentate nuclei (b), midbrain raphe nuclei (c), inferior colliculi (d), mammillary bodies (e), red nuclei (f), subthalamic nuclei (g), lateral geniculate nuclei (h), medial geniculate nuclei (i), and superior colliculi (j).

Supplementary Table 1. Visual assessment of ^{18}F-FDG PET images in each structure for each subject.

	Subject ID	**Iteration 4**	**Iteration 16**	**Iteration 64**	**Iteration 256**
**Inferior olivary nuclei**	1	poor	good	good	fair
	2	poor	good	good	good
	3	poor	good	good	good
	4	fair	good	good	good
	5	poor	good	good	good
	6	poor	fair	good	good
	7	poor	good	good	good
	8	poor	good	good	good
	9	poor	fair	good	good
	10	poor	good	good	fair
**Dentate nuclei**	1	fair	good	good	fair
	2	good	good	good	fair
	3	good	good	good	good
	4	good	good	good	good
	5	fair	good	good	fair
	6	fair	good	good	fair
	7	fair	good	good	fair
	8	good	good	good	fair
	9	fair	good	good	good
	10	good	good	fair	poor
**Midbrain raphe nuclei**	1	poor	fair	good	fair
	2	poor	fair	good	poor
	3	fair	fair	good	fair
	4	fair	good	good	good
	5	poor	fair	good	fair
	6	poor	fair	good	good
	7	poor	good	good	good
	8	poor	fair	fair	poor
	9	poor	fair	good	fair
	10	poor	good	good	poor
**Inferior colliculi**	1	good	good	good	good
	2	good	good	good	good
	3	good	good	good	good
	4	good	good	good	good
	5	good	good	good	good
	6	fair	good	good	good
	7	fair	good	good	good
	8	good	good	good	good
	9	fair	good	good	good
	10	good	good	good	good
**Mammillary bodies**	1	poor	fair	good	good
	2	poor	good	good	good
	3	poor	good	good	good
	4	poor	good	good	good
	5	poor	good	good	good
	6	poor	fair	good	good
	7	poor	good	good	good
	8	poor	good	good	good
	9	poor	fair	good	good
	10	fair	good	good	good

(Continued from supplementary Table 1)

	Subject ID	Iteration 4	Iteration 16	Iteration 64	Iteration 256
**Red nuclei**	1	good	good	good	fair
	2	fair	good	good	poor
	3	poor	good	good	fair
	4	poor	good	good	good
	5	fair	good	good	fair
	6	fair	good	good	fair
	7	fair	good	good	fair
	8	fair	good	good	fair
	9	fair	good	good	fair
	10	fair	good	good	fair
**Subthalamic nuclei**	1	fair	good	good	fair
	2	fair	good	good	fair
	3	poor	good	good	fair
	4	poor	good	good	good
	5	poor	good	good	fair
	6	poor	good	good	fair
	7	poor	good	good	fair
	8	poor	good	good	poor
	9	poor	good	good	fair
	10	poor	good	good	fair
**Lateral geniculate nuclei**	1	poor	good	good	poor
	2	poor	poor	good	poor
	3	poor	good	good	fair
	4	poor	fair	good	good
	5	poor	poor	good	fair
	6	poor	poor	good	poor
	7	poor	good	good	fair
	8	poor	good	good	fair
	9	poor	fair	good	fair
	10	poor	good	good	fair
**Medial geniculate nuclei**	1	poor	good	good	poor
	2	poor	poor	good	poor
	3	poor	good	good	fair
	4	poor	fair	good	good
	5	poor	poor	good	fair
	6	poor	poor	good	poor
	7	poor	good	good	fair
	8	poor	good	good	fair
	9	poor	fair	good	fair
	10	poor	good	good	fair
**Superior colliculi**	1	fair	good	good	poor
	2	poor	good	good	fair
	3	poor	good	good	poor
	4	poor	good	good	good
	5	poor	good	good	fair
	6	poor	fair	good	poor
	7	poor	good	good	poor
	8	poor	good	good	poor
	9	poor	good	good	good
	10	poor	good	good	fair

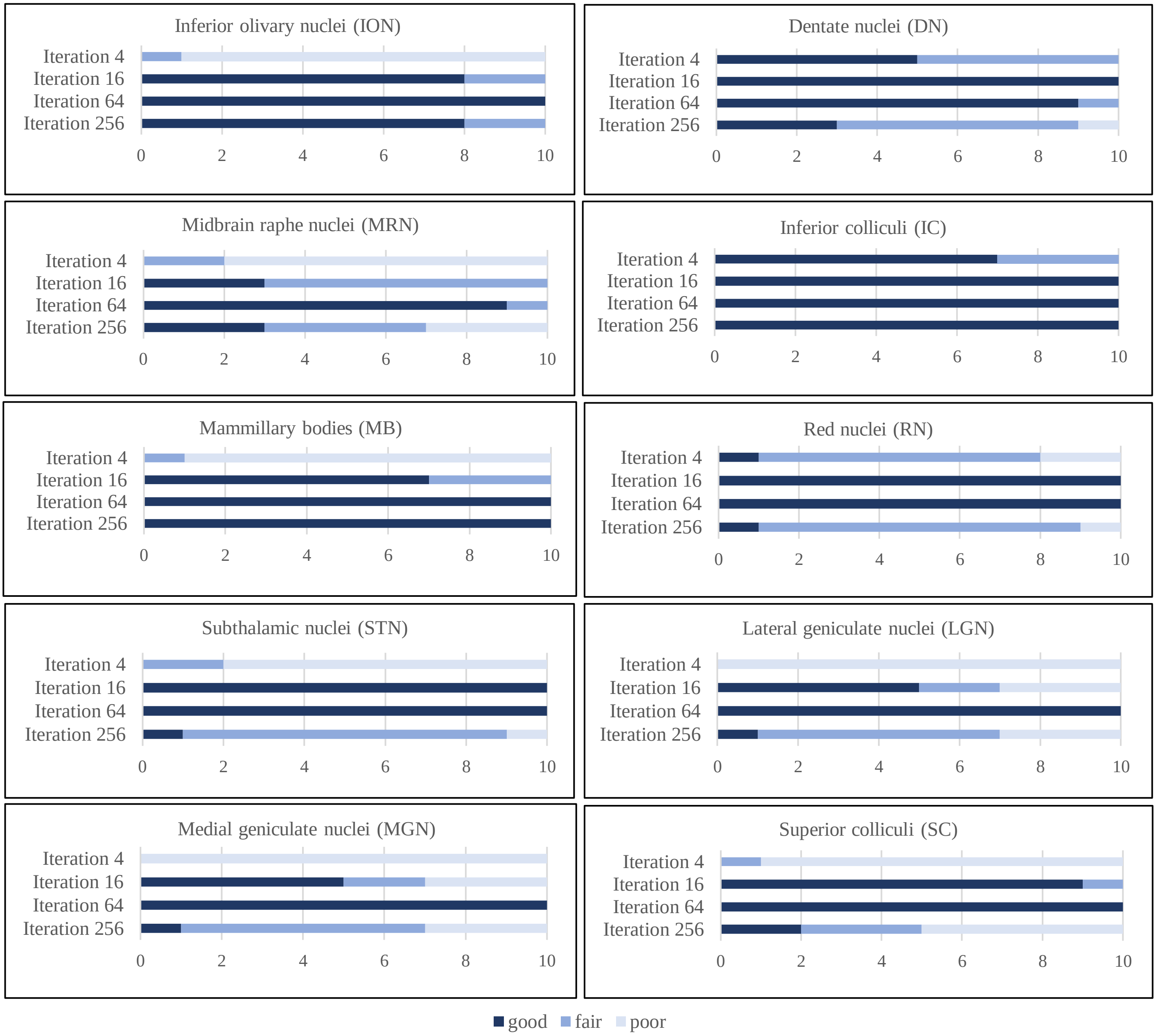

Supplementary Fig. 2. Bar chart of visual assessment of individual ^{18}F-FDG PET images in each structure for each iteration.

The ^{18}F-FDG PET findings in each structure for each iteration were as follows.

- ION: iteration 64, “good” in 10; iterations 256 and 16, “good” in 8 and “fair” in 2; iteration 4, “fair” in 1 and “poor” in 9.
- DN: iteration 16, “good” in 10; iteration 64, “good” in 9 and “fair” in 1; iteration 4, “good” in 5 and “fair” in 5; iteration 256, “good” in 3, “fair” in 6 and “poor” in 1.
- MRN: iteration 64, “good” in 9 and “fair” in 1; iteration 16, “good” in 3 and “fair” in 7; iteration 256, “good” in 3, “fair” in 4 and “poor” in 3; iteration 4, “fair” in 2 and “poor” in 8.
- IC: iterations 256, 64, and 16, “good” in 10; iteration 4, “good” in 7 and “fair” in 3.
- MB: iterations 256 and 64, “good” in 10; iteration 16, “good” in 7 and “fair” in 3; iteration 4, “fair” in 1 and “poor” in 9.
- RN: iterations 64 and 16, “good” in 10; iteration 256, “good” in 1, “fair” in 8 and “poor” in 1; iteration 4, “good” in 1, “fair” in 7 and “poor” in 2.
- STN: iterations 64 and 16, “good” in 10; iteration 256, “good” in 1, “fair” in 8 and “poor” in 1; iteration 4, “fair” in 2 and “poor” in 8.
- LGN&MGN: iteration 64, “good” in 10; iteration 16, “good” in 5, “fair” in 2 and “poor” in 3; iteration 256, “good” in 1, “fair” in 6 and “poor” in 3; iteration 4, “poor” in 10.
- SC: iteration 64, “good” in 10; iteration 16, “good” in 9 and “fair” in 1; iteration 256, “good” in 2, “fair” in 3 and “poor” in 5; iteration 4, “fair” in 1 and “poor” in 9.

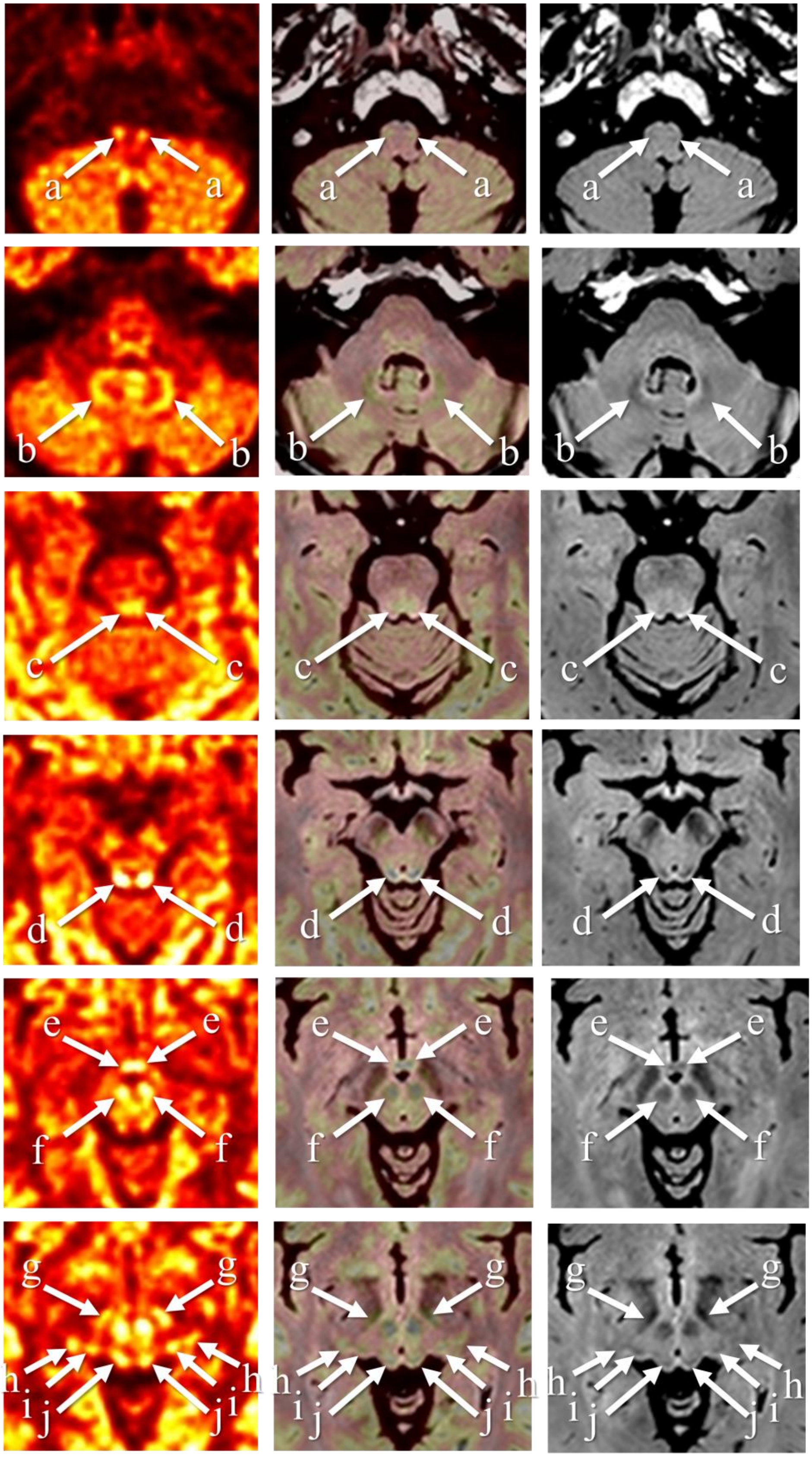

Supplementary Fig. 3. Zoomed ^{18}F-FDG PET (OSEM iteration 64 + TOF + PSF, left row), ^{18}F-FDG PET/3D-FLAIR fusion (middle row), and 3D-FLAIR images (right row) in a representative participant (subject ID = 9).

On the individual ^{18}F-FDG PET image with iteration 64, FDG uptake can be clearly distinguished in all structures, including inferior olivary nuclei (a), dentate nuclei (b), midbrain raphe nuclei (c), inferior colliculi (d), mammillary bodies (e), red nuclei (f), subthalamic nuclei (g), lateral geniculate nuclei (h), medial geniculate nuclei (i), and superior colliculi (j).
